# A Giant Arteriovenous Malformation and Fistula in a Newborn with Parkes Weber Syndrome. Case Report

**DOI:** 10.15388/Amed.2020.27.2.7

**Published:** 2020-12-23

**Authors:** Luize Auzina, Elina Skuja, Toms Janis Safranovs, Valts Ozolins, Helmuts Kidikas, Gita Taurina, Inguna Lubaua

**Affiliations:** Riga Stadins University, Riga, Latvia Children’s Clinical University hospital, Riga, Latvia; Riga Stadins University, Riga, Latvia; Riga Stadins University, Riga, Latvia; Children’s Clinical University hospital, Riga, Latvia; Paul Stadins Clinical University hospital, Riga, Latvia; Children’s Clinical University hospital, Riga, Latvia; Riga Stadins University, Riga, Latvia Children’s Clinical University hospital, Riga, Latvia

**Keywords:** Parkes Weber syndrome, RASA1, Neonate, Arteriovenous malformation, Port-wine stain

## Abstract

Parkes Weber syndrome (PWS) is a rare congenital condition characterized by capillary cutaneous malformation, limb hypertrophy and multiple arteriovenous fistulas of the affected extremity. Another feature is a port-wine stain on the affected area. PWS is caused by genetic variations in the RAS p21 protein activator (*RASA1*) gene which affects the development of the vascular system.

We report a case of a female neonate presenting with dyspnoea and cardiovascular insufficiency at the time of birth.

The left upper extremity (LUE) and shoulder were enlarged (circumference at the midpoint was 17 cm compared to 11 cm on the right arm), edematous, hyperemic with a port-wine stain. Structural changes of the bones of LUE were discovered on X-ray.

Echocardiography revealed right-sided volume overload, a large *ductus arteriosus*, a possible pathology of the aortic arch and branch arteries. Chest X-rays showed cardiomegaly. Therapy with milrinone and diuretics was started.

A multislice CT angiography scan revealed arteriovenous fistula (AVF) between *a. subclavia sin.* and* v. bra-chiocephalica sin, *arteriovenous malformations (AVM) and a dilated *a.subclavia sin.* of 11 mm, as well as dilatation of other arteries of the LUE. Next generation sequencing revealed a pathogenic variation (c.2245C>T, p.Arg749*) in the *RASA1* gene in the heterozygous state.

Four consecutive embolizations of the AVM and AVF were performed in the first 16 months.

## Abstract (lt)

**Didelis arterioveninis apsigimimas ir fistulė naujagimiui su Parkeso ir Weberio sindromu. Atvejo aprašymas:**

Parkeso ir Weberio sindromas (PWS) yra reta paveldima liga, kuriai būdinga odos kapiliarų mal-formacija, galūnių hipertrofija, taip pat dauginės arterioveninės fistulės ir vadinamosios portveino dėmės ligos pažeistoje galūnėje. PWS sukelia RAS p21 baltymo aktyvatoriaus (*RASA1*) geno variantas, kuris paveikia kraujagyslių sistemos vystymąsi.

Pristatomas klinikinis atvejis, kai gimusiam moteriškos lyties kūdikiui buvo dispnėja ir kraujagyslinis nepakankamumas.

Kairė ranka buvo padidėjusi, matoma nedidelė cianozė ir smulkios hematomos. Ranka ir petys buvo hi-peremiški ir edemiški, matomos portveino dėmės. Rentgenograma parodė esant struktūrinių VKG kaulų pa-kitimų. Daugiapjūvės KT angiografijos vaizduose matomos fistulės tarp* a. subclavia sin.* ir *v. brachiocephalica sin*. bei iki 11 mm išsiplėtusi *a. subclavia sin*., taip pat yra ir kitų VKG kraujagyslių išsiplėtimas.

Naujos kartos sekoskaitos metodu nustatytas patogeninis heterozigotinis *RASA1* geno variantas (c.2245C>T, p.Arg749*).

Per pirmuosius 16 mėnesių buvo atliktos keturios AVF ir AVM embolizacijos.

**Raktažodžiai**: Parkeso ir Weberio sindromas, *RASA1*, naujagimis, arterioveninės anomalijos, portveino dėmė

## Introduction

According to the classification by the International Society for the Study of Vascular Anomalies, PWS is defined as a complex combined of fast-flow vascular malformation that includes arterio-venous fistulas, capillary malformations associated with limb overgrowth and *RASA1* gene variations. [1] Capillary malformations are seen as warm, “geographic” pinkmacular skin lesion known as a port-wine stain on the affected limb [2, 3]. PWS can affect both the upper and lower extremities, however, it commonly affects one of the *lower* limbs. The condition may lead to cardiac overload, high-output cardiac failure, bleeding, thrombosis and ulceration [4]. These cases are mostly inherited in an autosomal dominant pattern from a symptomatic parent but they can also be sporadic due to a genetic variation in the *RASA1* gene encoding the p120-Ras GAP protein which is responsible for controlling cellular proliferation and differentiation [5, 6].

In this case report, we present a neonate that initially presented with an enlarged upper extremity and cardiovascular insufficiency right after birth. The aim of this case report is to present a successful multistage embolization in a PWS patient and briefly diferenciate the diagnosis of a complex vascular malformations

## Clinical course

A new-born girl was admitted to the Neonatal Intensive Care Unit (NICU) at Children’s Clinical University Hospital with suspected congenital heart disease on the day of birth. The patient was born at 39 [4, 7] weeks of gestation to a 32 year-old mother from the 1st pregnancy by spontaneous vaginal delivery. The birth weight was 3650 g, height 50 cm, and Apgar score 7/8. She presented with labored breathing and was started on infant T piece resuscitator “NeoPuff” (SpO_2_ at the 10^th^ minute of life was 98%). The left upper extremity (LUE) and shoulder were enlarged, severely oedematous, mildly cyanotic, and hyperaemic with a “geographic” port-wine stain; the circumference of the patient’s left upper arm’s midpoint was 17 cm compared to 11 cm on the right upper arm. There was a localized hyperthermia and thrills over the LUE. Patient was tachypnoeic and had signs of volume overload. Mild systolic heart murmur was heard over the left sternal border.

**Figure 1.**
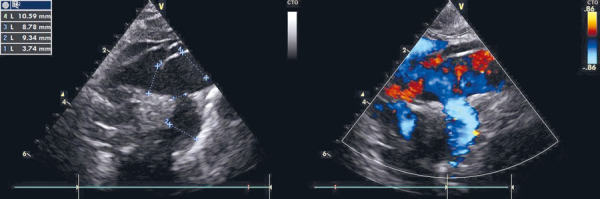
Echocardiography after birth showing a dilated a. subclavia sinistra

Echocardiography revealed right-sided volume overload and raised suspicion of a possible anomaly of the aortic arch and cervical blood vessels and a possible arteriovenous malformation (Figure 1). 

The aortic arch and *a. subclavia sinistra* were dilated, there was a retrograde diastolic flow in the descending aorta. The *ductus arteriosus* was open. In order to clarify the diagnosis, cardiac CT was assigned.

A chest and abdominal X-ray showed cardiomegaly (CTI- 0.74) and tissue thickening of the neck and the left side of the thorax, cystic lesions, overgrowth and structural changes in bones of the LUE. 

Therapy with milrinone (0.5 µg/kg/min), furosemide (1 mg/kg) twice daily, and spironolactone (1 mg/kg) twice daily was started. Milrinone was discontinued on the 5^th^ day, while diuretics were gradually weaned and discontinued after four weeks.

**Figure 2.**
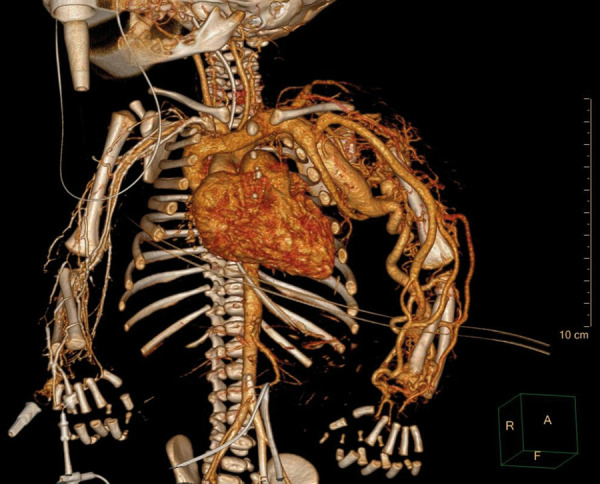
CT angiography showing AV malformations in the left scapula and shoulder region and AV fistulas between *a. subclavia sin.* and *v. brachiocephalica sin*.

On the second day of life a multislice CT angiography scan revealed a dilated *a. subclavia sin.* 11 mm in diameter (+4,81 Z score) as well as dilatation of other arteries of the LUE. *V. cava superior* (13 mm) and *v. brachiocephalica *(9 mm) were also dilated. A fistula between *a. subclavia sin.* and** *v. brachiocephalica sin. *3.5 mm wide was seen, as well as AV malformation in the left scapula and shoulder region (Figure 2). Magnetic resonance imaging (MRI) angiography was also considered, but due to the unstable clinical condition CT angiography was preferred.

The* ductus arteriosus *remained wide open (2.8 mm) until the 18^th^ day, then it started to shrink.

**Figure 3.**
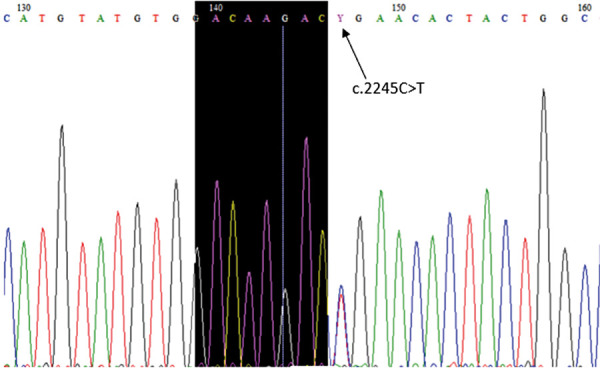
*RASA1* gene variant (c.2245C>T, p.Arg749*) in the heterozygous state

Next generation sequencing was performed, and at the age of two months results revealed a *RASA1* gene variant (c.2245C>T, p.Arg749*) in the heterozygous state (Figure 3). The variant has been previously reported in at least two patients with capillary–arteriovenous malformation (CM– AVM), therefore the variant has been classified as pathogenic [4].

After 9 days the patient was transferred from the NICU to the neonatal ward, and at the age of 25 days the patient was discharged with no signs of cardiovascular insufficiency. The therapy was discontinued before discharge.

**Figure 4.**
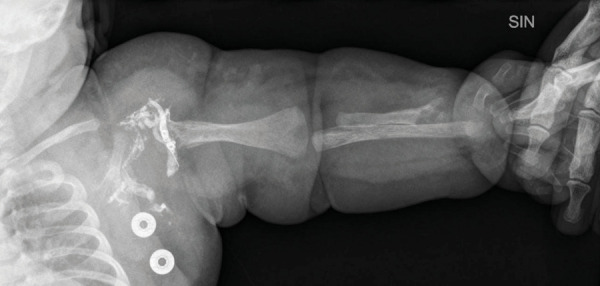
An X ray image of the left arm showing structural changes of the bones (5 weeks old, after the first embolization)

**Figure 5.**
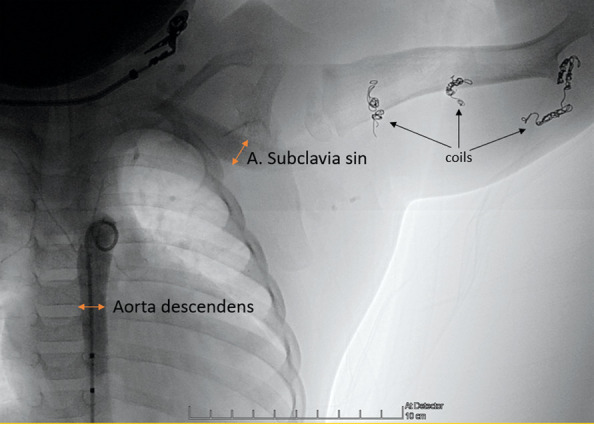
Descending aorta angiography before the fourth procedure. The diameter of the dilated *a. subclavia sinistra* is the same as the descending aorta. Multiple coils (black arrows) in different locations in the left arm after the previous embolizations.

**Figure 6.**
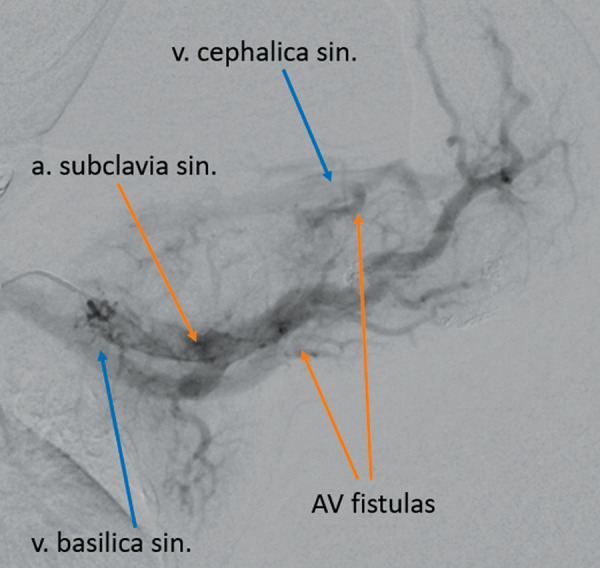
AV fistulas visible between *a. sublavia sinistra* and *v. cephalica sinistra*, and *v. basilica sinistra* during the selective left subclavian artery angiography

**Figure 7.**
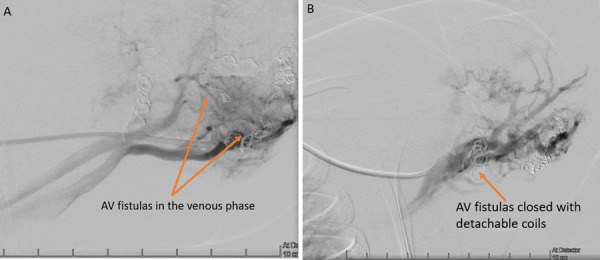
A – AV fistulas visible in the venous phase of the *a. subclavia sinistra* angiography. B – AV fistulas closed with detachable coils.

**Figure 8.**
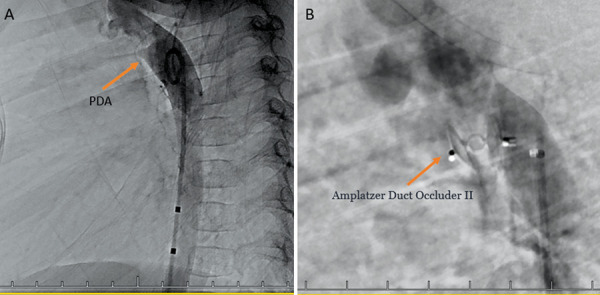
A – PDA visible during the aortography. B – PDA closed with Amplatzer Duct Occluder II

The patient underwent arteriovenous embolization four times – at the age of 5 weeks, 5 months, 10 months and 16 months. After the second embolization, the heart murmur could no longer be heard and a thrill was no longer palpable over the armpit. Staged embolization of arteriovenous fistulas and malformations were done under general anesthesia using the femoral artery approach. With the support of a 4 Fr diagnostic catheter, selective catheterization of high flow arteriovenous fistulas using a microcatheter was done with subsequent embolizations. Detachable micro-coils were used in the first three embolisations to reduce the flow in the arterial feeder, and several fistulas were closed by additional injection of *Histoacryl* glue (30% cyanoacrylate/lipiodol mixture). An X-ray was done after the first embolization (Figure 4). During the fourth procedure selective angiography of the descending aorta and left subclavian artery still revealed dilatation of the left subclavian artery and multiple fistulas between *a. subclavia sinistra* and *v. cephalica sinistra* (Figures 5 and 6) One was closed with *Histoacryl* glue and the two others with coils (Figure 7). Additionally, the patent *ductus arteriosus* was closed with an Amplatzer Duct Occluder II (Abbott) (Figure 8).

*Luize Auzina et al. *A Giant Arteriovenous Malformation and Fistula in a Newborn with Parkes Weber Syndrome. Case Report

**Figure 9.**
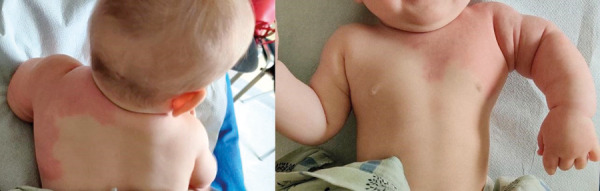
Edema and a port-wine stain on the left upper arm (8 months old).

**Figure 10.**
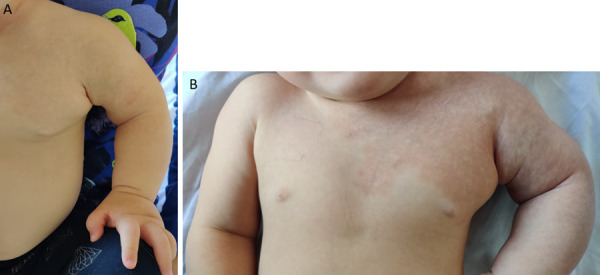
Left arm aft er the fourth procedure at the age of 16 months. A – Patient is calm and sitting (skin appearance of the left arm almost normal). B – Patient crying in the supine position (port-wine stain more pronounced)

During follow-up the circumference of the left upper arm was increased, as the child grow. It was 19.5 cm after the first embolization and 20 cm after the second embolization compared to 13 cm and 14 cm, respectively, for the right arm. At the age of 8 months, the port-wine stain was less intense and appeared mostly in the horizontal position (Figure 9). After the fourth procedure at the age of 16 months, the left arm became less edematous and hyperaemic. Nevertheless when the patient is crying or is in supine position hyperemia and the port-wine stain became visible (Figure 10). The diameter of the left upper arm after the fourth procedure was 21 cm, while the right upper arm was 16 cm in diameter.

The patient wears a compression garment daily and visits the physiotherapist regularly. The patient’s motor development has improved significantly over time. The functions, muscle force and extent of movement in the affected limb is almost equal to the right arm at the age of 16 months. There are no signs of heart failure and the patient is taking no medications.

Genetic testing was performed for both parents and the same genetic variation was revealed in one of them. Th e parent is asymptomatic, but a head MRI has been planned to reveal a possible vascular or lymphatic anomalies. 

## Discussion

Parkes Weber syndrome (PWS) was first described by Frederick Parkes Weber, a British dermatologist, as a “vascular lesions with hemihypertrophy” [7, 8].  

According to the classification published by the International Society for the Study of Vascular Anomalies, PWS is defined as combined capillary malformation (CM), fast flow arteriovenous malformations (AVM) and arteriovenous fistulas (AVF) associated with limb overgrowth [1, 3].**Warm, pink to red macular skin lesions known as a port-wine stain or pseudo capillary malformation can also be seen on the affected limb [3, 9].**Other common signs include lymphoedema and lymphatic vesicles that can be seen on the skin. Varicosities form as a result of fast flow shunting in the AVF [9].

AVM is a high-flow anomaly characterized by a precapillary arteriovenous shunt, called ‘nidus’ which occurs due to developmental defects during various stages of embryogenesis [10, 11, 12]. AVFs are also high-flow vascular malformations consisting of precapillary shunts but no nidus are seen [12].

PWS is mostly seen in lower extremities, however it can affect both upper and lower extremities. In this particular case, the left upper limb was affected. The mechanism of limb overgrowth is still unclear, but most likely it can be explained through physiological processes. As a result of tissue hypoxia, due to the steel phenomenon created by the AVFs and release of angiogenic growth factors, neovascularization of the cartilaginous growth centers is increased. AVFs can occur anywhere in the body including cutaneous, subcutaneous, intramuscular, intraosseous, and cerebral regions [3, 13].

PWS is frequently misdiagnosed as Klippel-Trenaunay syndrome (KTS), which is characterized as a triad of capillary, venous and lymphatic malformations associated with limb overgrowth, but without arteriovenous fistulas, as it is in PWS [3]. That is why cardiac failure due to volume overload is not typical in KTS patients as opposed to PWS patients. The vascular skin stain in PWS patients seen on the affected limb could be described as “pseudo CM” (not a real CM as it is for KTS), because they are composed of micro AV fistulas which could explain the warmth (high flow in the AV fistulas). Patients with PWS are at risk of ulceration, cardiac overload and high-output cardiac failure, especially in the neonatal period if there are multiple AVFs**[4, 9].

PWS is caused by genetic variations of the *RASA1* gene located on the chromosome 5q13.1, which encodes the p120-Ras GAP protein. This protein promotes signaling through the tyrosine kinase pathway for various growth factor receptors that control proliferation, migration and survival of several cell types, including vascular endothelial cells [5, 7]. PWS is inherited in an autosomal dominant manner [6].

Genetic counseling of the patient’s family revealed no other possibly affected family members, nor did it reveal sudden nor early deaths. This could cause a failure to recognize the disorder in family members, thinking that the disease might be not inherited. Genetic testing in patients with congenital capillary arteriovenous malformations should be performed in order to reveal the possible inheritance of disease and, of course, for family planning. In case a pathogenic variant is found in the patient, seemingly healthy parents must be tested in order to avoid possible disease complications. Although there is different literature data, the disease penetrance is up to 90–99%, which suggests that although parents are so far asymptomatic, they might be symptomatic later in life. The most important malformation to rule out is a Galen vein aneurysm. Targeted questions should be asked to the family with congenital AVM regarding family history, such as history of headaches and migraines, as well as skin conditions, looking for skin discoloration and discrepancy in size of extremities. 

To assess the location and extent of AVMs, it is important to carry out a proper diagnostic imaging. Doppler ultrasound can be used as an initial evaluation to distinguish fast flow AVMs from slow flow vascular malformation. Computed tomography is more suitable than magnetic resonance imaging for any arterial or venous abnormalities in newborns and infants, especially if the blood vessels are approximately a millimetre in size. Radiation exposure is, of course, a concern in paediatric imaging, but diagnostic quality remains the most important factor [14]. MRI angiography is also used to reveal musculoskeletal abnormalities and vascular malformations, such as engorged arteries, veins, capillaries and flow voids, as well as arteriovenous fistulas [3]. During cardiac MRI neonates need to be under general anesthesia and often require myorelaxants and induced apnoea. An MRI study can take up to 45-60 minutes and is considered unsafe for patients in unstable clinical conditions. Additionally for this pathology high spatial resolution is needed, and CT is better compared to MRI [15]. 

During follow-up of patients presenting with cardiac symptoms and overgrowth, issues must be monitored. The progress of the disease may be slow and not predictable early in life. The majority of patients have some degree of pain, swelling, overgrowth, and nerve compression at some anatomic level [16].

A complete cure of the disease is not possible. The main goal is to obtain an acceptable quality of life for the patient. The main aims of the treatment are to slow the disease progression, prevent heart failure, limb hypertrophy and development of varicose veins. Pharmacologic treatment is not effective in these cases. Compression garment therapy is used as a supportive method. Treatment for AVMs and AVFs is similar. Embolization is considered as a preoperative approach but may also be used as definitive treatment. Almost always repeated embolizations over many years are necessary and there is no fast and definite way to treat peripheral AVMs and AVFs [12]. In our case multistage embolization was chosen and showed promising result. Isolated embolization is recommended in nonsurgical candidates who present with multiple deep AVFs [5]. The rate of successfully controlled symptoms is up to 70% using selective embolization [16]. Surgery is safe and well tolerated in PWS patients with a stable clinical condition. For localized cutaneous AVFs, surgical resection is indicated. In individual cases, spontaneous regression of a limb AVM in PWS has been described [5].

## Conclusion

In this case report, we described a patient with genetically confirmed PWS with a pathogenic genetic variation**in the *RASA1* gene. The patient showed typical signs for PWS, such as a port-wine stain, limb overgrowth arteriovenous malformations and an arteriovenous fistula. 

The limitations of this study is the uniqueness of the case. This is the first patient with genetically confirmed PWS in Latvia and there is no data in our clinic to compare with and evaluate the treatment plan and estimate long term prognosis.
